# A novel microfluidic 3D platform for culturing pancreatic ductal adenocarcinoma cells: comparison with *in vitro* cultures and *in vivo* xenografts

**DOI:** 10.1038/s41598-017-01256-8

**Published:** 2017-04-25

**Authors:** Meike Beer, Nirmala Kuppalu, Matteo Stefanini, Holger Becker, Ingo Schulz, Sagar Manoli, Julia Schuette, Christian Schmees, Armando Casazza, Martin Stelzle, Annarosa Arcangeli

**Affiliations:** 1 NMI-Natural and Medical Institute at the University of Tuebingen, Tübingen, Germany; 2 Department of Experimental and Clinical Medicine, University of Florence, Florence, Italy; 3 Dival Toscana Srl, Sesto Fiorentino, Italy; 4 Microfluidic ChipShop, Jena, Germany

## Abstract

The integration of microfluidics and cell biology has reached a significant milestone with the development of “organ-on-chips”, smart technological platforms that, once applied to the study of human diseases, such as cancer, might ultimately contribute to design personalised treatments and hence improve health outcomes. This paper reports that the combination of microfluidics and dielectrophoresis (DEP) allows to culture different pancreatic ductal adenocarcinoma (PDAC) human cell lines into a cyclic olefin polymer (COP) chamber (HepaChip^®^), enriched by the extracellular matrix (ECM) protein collagen. We show that PDAC cells cultured into the HepaChip^®^ (1) are vital and grow, provided they properly attach to collagen; (2) show morphological appearance and growth characteristics closer to those of cells grown as spheroids than as classical 2 dimensional (2D) *in vitro* cultures. Finally, preliminary experiments show that PDAC cells respond to high doses of Cisplatin perfused through the chip. Overall, the present microfluidic platform could be exploited in the future for a personalised approach to PDAC.

## Introduction

The deciphering of tumour biomolecular features and growth dynamics and the identification of novel targeted therapeutic strategies is being one of the major challenges in oncological research. Different platforms, based on either *in vitro* cell cultures in two dimension (2D) or *in vivo* animal models, have been proposed and employed for pre-clinical testings^1, 2^. While *in vitro* 2D cultures have been the cornerstone of pre-clinical cancer research, there is increasing evidence that cells grown in 2D monolayers do not accurately reflect the biological complexity of tumours. In particular, they lack the complex extracellular matrix (ECM)-cancer interactions as well as intra-tumoral gradients in pH, oxygen and nutrients, which have been found in cancers *in vivo*. These weaknesses can explain why many drugs, that pass pre-clinical *in vitro* testing, fail in the patients^3–6^. On the other hand, “classical” *in vivo* pre-clinical mouse models, e.g. subcutaneous or orthotopic xenografts of human tumour cells in immuno-compromised mice, poorly recapitulate the proper tumour behaviour and undermine the impact of the tumour microenvironment, in particular of acquired immunity. Moreover, animal models are expensive, time consuming and under some aspects “non-ethical”^4, 5^.

Scientists hence realised the necessity of using more complex three dimensional (3D) cell cultures for better understanding tumour characteristics in a proper microenvironment and testing the responses to different drugs. Generally, 3D cultures can more closely mimic physiological conditions over 2D monolayers, as they more accurately reflect the architecture and bio-mechanical properties of the tumour tissue. In addition, 3D cultures can reproduce several parameters of tumour microenvironment, including oxygen and nutrient gradients as well as the development of a dormant tumour region^7, 8^. Overall, 3D cultures allow to monitor cell growth dynamics and response to treatments more appropriately, and could hence fill the gap between *in vitro* and *in vivo* systems for preclinical oncological research. As a result, there has been increasing focus in developing 3D techniques, and many different platforms have been proposed, with different grades of complexity and expression of tumour microenvironmental conditions^5, 9^.

A further improvement in this field could derive from the integration of microfluidics and cell biology, which has recently reached a significant milestone with the development of “organ-on-chip” technologies. What began at the turn of the millennium as simple demonstrations of biological cells being transported and manipulated in microchannels for basic short-term analysis, has now advanced to the point where we can engineer living cellular microsystems with controllable microenvironments that behave and function – with organ-level complexity – like their counterparts *in vivo* ^10–14^. Organ-like features include continuous perfusion and physiological cell–matrix and cell–cell interactions. More recently, the “organ on chip” technology has been transferred to study human disease models, including cancer^15–20^. As a further step, the combination of microfluidics and dielectrophoresis (DEP) to assemble primary human cells, has enabled the automated *in vitro* construction of micro-organs, which mimic proper *in vivo* structures. As a unique feature of organ-on-chip technology, the use of DEP selectively assembles only viable cells^21, 22^. For the HepaChip^®^ organ-specific 3D cell culture chambers are designed and validated by multiphysics simulations and realised by injection moulding of cyclic olefin polymer (COP)^21, 23, 24^. Proprietary surface functionalization enables selective deposition of ECM proteins in a simple perfusion process^25^. High resolution optical imaging of micro-organs along with the complete set of staining technologies is possible due to the exceptional optical properties of COP.

We applied these concepts to create a novel platform for studying pancreatic ductal adenocarcinoma (PDAC), one of the human cancers with worst prognosis, for which the design of novel therapeutic options is urgently needed. For these reasons, various model systems are being developed, from *in vitro* 2D and 3D cell cultures, to whole animal models^26^.

We here provide evidence that human PDAC cells can be cultured onto a novel microfluidic chamber, the HepaChip^®^, maintaining cell vitality and displaying appropriate morphological appearance, growth characteristics and response to chemotherapeutic drugs.

## Materials and Methods

### Cell lines

The PDAC cell lines; PANC1, BxPC3 and MiaPaCa2 were used for the experiment. The Panc1 and MiaPaCa2 cells harbour mutation in KRAS and TP53, homozygous deletion (HD) in CDKN2A/p16 and wild type (WT) SMAD4, while BxPC3 cells harbour mutation in TP53, HD in SMAD4 and WT KRAS^27^.

### Two dimensional cell culture (2D)

PANC1 and MiaPaCa2 cells were cultured using Dulbecco’s Modified Eagle Medium (DMEM) supplemented with 10% foetal bovine serum (FBS) (Hyclone, SH30070.03) and 4 mM glutamine. BxPC3 was cultured using RPM1-1640 medium (EuroClone) supplemented with 10% FBS (Hyclone, SH30070.03) and 2 mM glutamine (Sigma). Cells were cultured in a humidified 5% CO_2_ at 37 °C.

#### Growth Curve and Live/Dead cell imaging

Ten thousand cells were seeded in each well of a 96 well plate in 200 µL of media. Cell density was estimated every 24 h by manual counting in the presence of trypan blue dye to exclude dead cells. After 48 h of culturing cells were stained with Calcein AM (2 µg/mL) (Molecular Probes) and Propidium Iodide (PI) (10 µg/mL) (Sigma) for 20 minutes. Then washed with Phosphate-Buffered Saline (PBS) and Images were taken using Nikon Eclipse TE300 florescence microscope.

#### Phalloidin staining

Thin glass cover slips were ethanol and flame sterilised. The cells were seeded on the glass cover slips for 24 h. The unattached cells were washed away with PBS and cells were fixed with 4% methanol-free formaldehyde (Thermo scientific) for 20 minutes at room temperature. Cells were permeabilised with 0.01% Triton-X for 5 minutes at room temperature, washed with PBS. Nonspecific sites were blocked using 10% BSA for 1 h. Then cells were stained using rhodamine conjugated Phalloidin (Sigma), followed by nucleus staining using 4′,6-diamidino-2-phenylindole (DAPI; Invitrogen). Images were captured using confocal microscope (Nikon Eclipse TE2000-U).

#### Drug Treatment and analysis of drug efficacy

Ten thousand PANC1 cells were seeded per well (96 well plate). After 24 h of culturing, cells were treated with Cisplatin (Sigma) of 0, 1, 2.5,5,10,25,50 and 100 µM concentration. After 24 h of drug treatment, cells were detached using trypsin and counted manually using trypan blue as above.

### Three dimensional cell culture (3D)

Three dimensional spheroids were generated using liquid overlay technique (U tube technique). Cells were treated with trypsin and counted using trypan blue. Subsequently cells were seeded onto agarose-coated (50 µL, 1.5% agarose/well) 96 well plate in 100 µL of medium^28^. After 72 h, 100 µL medium was added and afterwards, every 48 h, 50% of the supernatant was replaced with fresh medium. Spheroids were cultured at 37 °C in a humidified atmosphere containing 5% CO_2_ in air.

#### Growth Curve and Live/Dead cell imaging

Five hundred cells were seeded on agarose coated 96 well plate. Spheroids were imaged using bright field microscopy (Nikon) every day with a 10x objective. Image resolution was 0.757 µm/pix. Spheroid volume was calculated using SpheroidSizer program as reported by Chen W. *et al*.^28^. After 72 h of cell seeding, cells were stained by adding fresh medium containing Calcein AM (2 µg/mL) and PI (10 µg/ mL), incubated for 20 minutes and then washed with PBS. Images were taken using Nikon Elipse TE300 florescence microscope.

#### Phalloidin staining

After 72 h of culturing, spheroids were fixed with 4% formaldehyde, then permeabilised using 0.01% Triton-X for 5 minutes, and washed with PBS. Cells were then blocked using 10% BSA for 1 h, and then stained using rhodamine conjugated Phalloidin. Nucleus was stained using DAPI. Images were captured using confocal microscope (Nikon Eclipse TE2000-U).

#### Drug Treatment and analysis of drug efficacy

For drug treatment, 1000 PANC1 cells were seeded per well (96well plate). After 72 h, spheroids were treated with Cisplatin of 0, 5,10,20,50 and 100 µM concentration. Untreated spheroids were also cultured in parallel. Spheroids were maintained without changing medium for the whole experiment. After 72 h of drug treatment, 20 to 30 spheroids were collected, washed with PBS, dissociated using 5 mM Ethylenediaminetetraacetic acid (EDTA) and incubated with PI (0.05 µg/mL) for 5 min. Stained cells were analysed using flow cytometer.

### Microfluidic cell culture device (HepaChip^®^)

The device technology was previously described^21, 23, 24^. Briefly, the device comprises eight identical cell chambers, containing three cell culture regions each, sized 1 mm × 60 µm, i.e. 24 cell culture regions per chip. These culture regions were selectively coated with collagen to facilitate cell adhesion as reported before^25^. In brief, the regions were irradiated through a shadow mask by UV light to induce acid groups on the surface. Collagen was incubated from a 20 µM solution for 90 min and bound to these acid groups. After rinsing the chip was sealed with an adhesive foil (polyolefin cover foil, HJ-Bioanalytik Moenchengladbach). Chips (without cover foil) were manufactured by microfluidic ChipShop, Jena, by micro injection moulding.

On the side walls of each cell chamber electrodes are integrated which are used to generate an inhomogeneous high frequency electrical field. This gives rise to dielectrophoretic forces by which cells entering the cell chamber are drawn into the assembly areas. Only viable cells will be selectively assembled as only they exhibit intact cell membranes with their intracellular permittivity differing from that of the extracellular medium they are suspended in.

The microfluidic devices were mounted in a fixture providing for fluidic, electrical, and thermal contact and control of the chip by an external periphery unit containing Polytetrafluoroethylene (PTFE) tubes and syringe pumps as well as function generator, amplifier and electronic heat control.

#### *Cell assembly and culture in HepaChip*^®^

Cells were suspended in a DEP medium exhibiting an especially low conductance of 80 to 200 µS/cm. It comprised 95 g/L sucrose, 1 g/L glucose, 57.9 mg/L sodium pyruvate, 28.4 mg/L calcium chloride and 24.6 mg/L MgSO_4_ (aq). From this suspension cells were assembled by DEP at a flow rate of 100 µl/min, a peak to peak voltage between 140 and 150 V and a frequency of 350 kHz, for durations of 3 to 5 min.

After cell assembly, culture medium was perfused through the chip for 3 to 9 days at a perfusion rate of 3.125 µl/min. During culture the chips were maintained at temperatures between 35 °C and 37 °C while the medium reservoirs were heated to between 37 °C and 39 °C. To make sure that no gas or air bubbles enter the microfluidic chips, a bubble trap was inserted in the inlet tubing of each chip. In this trap the medium flows underneath a gas permeable PTFE membrane where bubbles can exit but the medium is kept inside the system.

#### *Viability assay in HepaChip*^®^

Each day the cells in the microfluidic chip were stained with green fluorescent Calcein AM (2 µg/mL) and red fluorescent PI (10 µg/mL) in order to visualise viable cells in green and nuclei of dead or dying cells in red. After incubating with the staining solution for 20 min at 7 µL/min the chip was washed with culture medium before the perfused culture restarted. Images were captured with Nikon DS 2MBWc microscope camera using Nikon Eclipse Ti.

#### *Phalloidin staining in HepaChip*^®^

The chip was flushed with 4% paraformaldehyde for 20 min at 15 µL/min to fix the cells. Next the cells were permeabilised by pumping phosphate buffered saline (PBS) with 1% triton X through the chip for 10 min at a flow rate of 50 µL/min. Then cells were stained with green fluorescent Phalloidin and DAPI in PBS for 20 min at 15 µL/min and finally washed with PBS at 50 µL/min. The cells in the chips were then imaged as those stained in the viability assay.

### *In vivo* experiments on nu/nu mice

*In vivo* experiments were performed at Dival Toscana Srl, inside the Animal Facility of the University of Florence. All experiments on live vertebrates were performed in accordance with relevant guidelines and regulations. We confirm that the Ethical Committee of the University of Firenze approved all the experiments described in this paper. Female, 9 weeks aged, athymic nude nu/nu mice (Envigo-Harlan Italy; Udine) were subcutaneously (s.c.) injected with 3 × 10^6^ PANC1 cells per mouse. Mice were treated daily intraperitoneum (i.p.) with Cisplatin (10 mg/kg) (n = 4 masses) or with saline (n = 4 masses) for one week, starting the day the masses reached the measure of (0,2 × 0,2 cm). Tumour growth was monitored daily by measuring two perpendicular diameters; the volume of the tumour masses was calculated using the ellipsoid equation. At the end of the treatment, all mice were sacrificed, and tumour masses were collected measured and weighted.

### Statistical analysis

Data are generally given as mean values ± standard error of the mean (SEM), with n indicating the number of independent experiments. When the mean ± standard deviation (SD) was calculated, it is indicated in the figure legend. Statistical comparisons between two groups of data were performed with the Mann-Whitney test.

## Results

**Figure 1:**
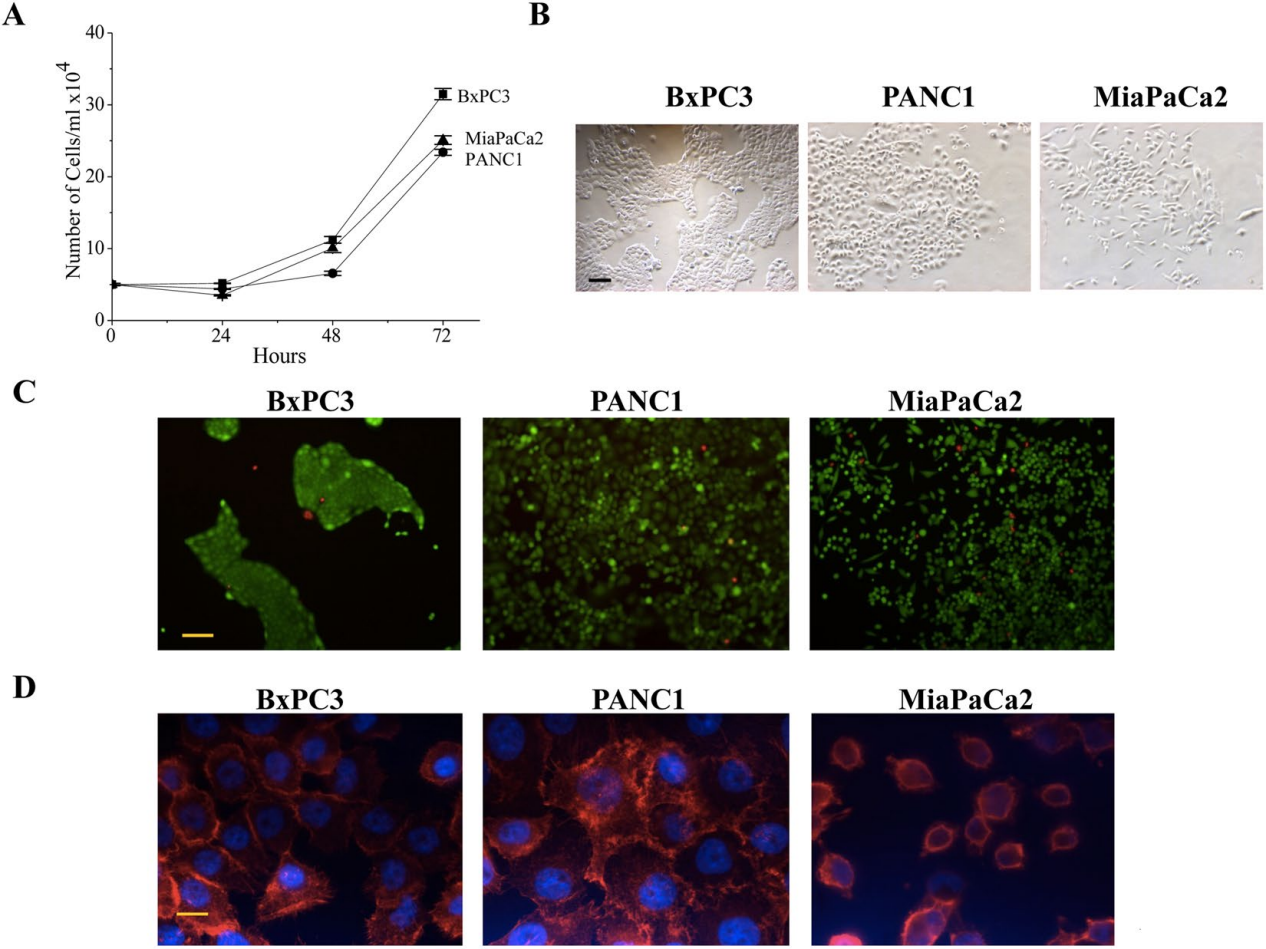
*In vitro* cell growth characteristics of PDAC cells in 2D culture conditions. **(A)** Comparison of growth rate among the PDAC cell lines in terms of increase in number of cells per day (n = 3). Data are means ± SEM of three separate experiments each carried out in triplicate. At the 0.05 level, the PANC1, MiaPaCa2 and BxPC3 are not significantly different. **(B)** Microscopic images after 48 h of culturing of PDAC cell lines. Morphology was different among the cell lines. **(C)** Live and dead cells were stained by Calcein (Green) and PI (Red) respectively after 24 h of culturing. **(D)** Actin filaments were stained by rhodamine conjugated Phalloidin (Red) and nucleus was stained by DAPI (Blue). Scale bars in **B** and **C** represent 100 µm, in **D** represents 10 µm.

**Figure 2:**
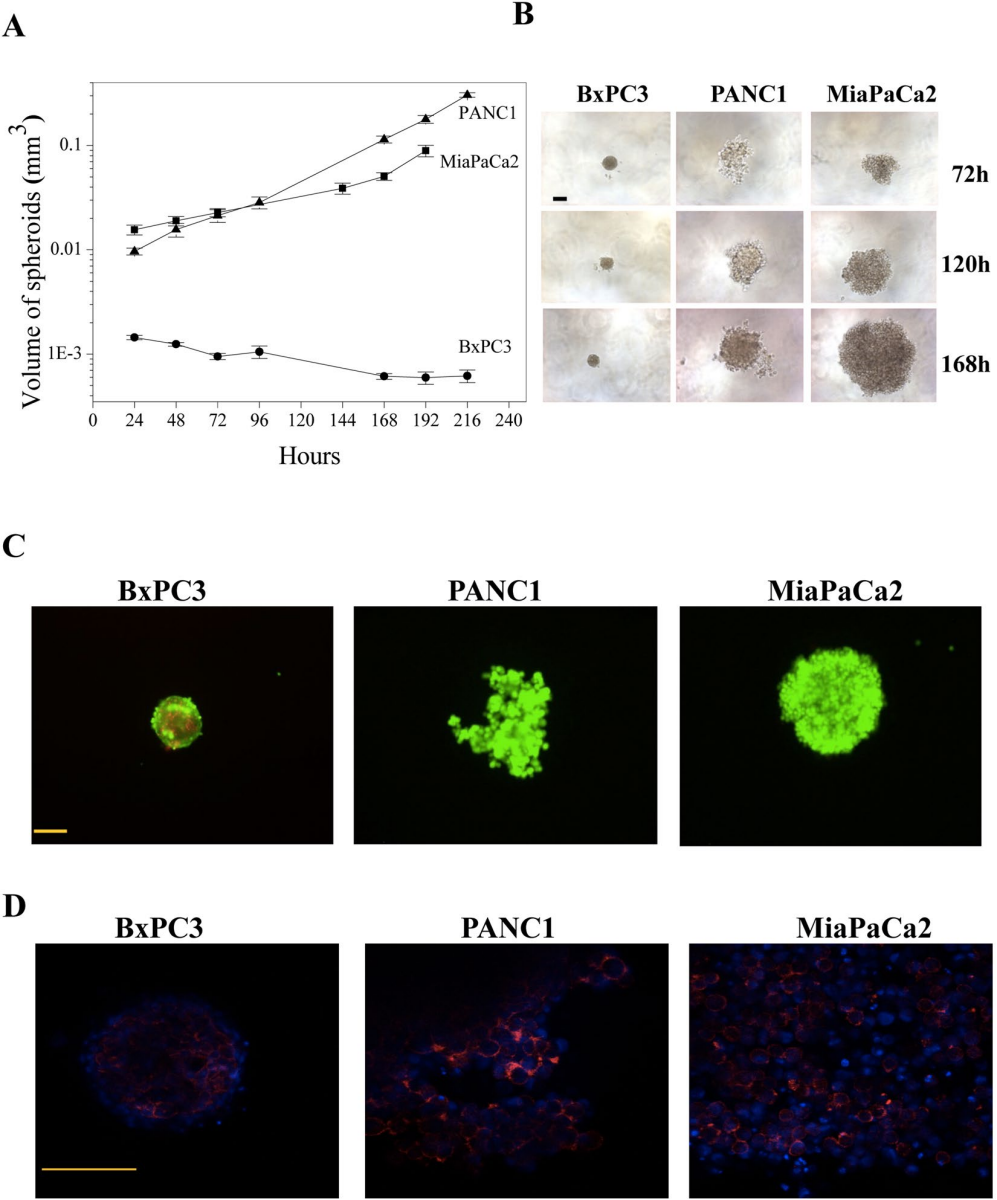
*In vitro* cell growth characteristics of PDAC cells in 3D culture conditions. **(A)** Comparison of growth rate between the PDAC cell lines with respect to difference in spheroid volume per day (n = 3). Data are means ± SEM of three separate experiments each carried out in triplicate. At the 0.05 level, data relative to MiaPaCa2 and PANC1 are not significantly different, whereas data relative to MiaPaCa2 are significantly different from those relative to BxPC3 (p = 0.003), and data relative to PANC1 are significantly different from those relative to BxPC3 (p = 0.002). **(B)** Microscopic images of PDAC at 72 h (top layer), 120 h (middle layer) and 168 h (bottom layer). **(C)** Live and dead cells were stained after 72H of culturing by Calcein (green) and PI (red) respectively. **(D)** Cytoskeleton organisation was studied by actin staining in all the three cell lines found to be on the periphery of cells. Scale bars represent 100 µm.

The aim of the present study was to demonstrate the feasibility of obtaining healthy and reproducible cultures of PDAC cells in a microfluidic chamber, which enables active assembly of cell aggregates and cultivation under continuous and controlled perfusion. For this purpose, we used three different human PDAC cell lines (BxPC3, PANC1 and MiaPaCa2), whose growth characteristics were first analysed in 2D (Fig. 1) and spheroid 3D (Fig. 2) cultures. In 2D culture conditions, all the PDAC cells proliferate roughly at the same extent and with similar kinetics, reaching a plateau at about 3 × 10^5^ cells/ml after 72 hours of incubation (Fig. 1A). As evident from phase contrast pictures in Fig. 1B, BxPC3 cells show a flattened morphology, with the tendency to grow as small “islands”. MiaPaCa2 cell cultures show two types of cells (1) Adherent cells with a mesenchymal morphology, with round body and more or less long and substrate adherent cytoplasmic extensions and (2) Round, less adherent cells. PANC1 display an intermediate morphology, not fully epithelial, more mesenchymal, but more adherent to the substrate compared to MiaPaCa2 cells. Roughly all the PDAC cells in 2D cultures are vital after 48 hours of incubation, as witnessed by the Calcein/PI staining (Fig. 1C). One of the functional characteristics of cancer is their propensity to migrate, an essential step for further metastatisation. Hence cancer cells are characterised by a peculiar organisation of their actin cytoskeleton which can underlie the formation of lamellipodia^29^. We then chose to determine the state of the actin cytoskeleton, determined by the Phalloidin staining, in the different PDAC cell lines as a functional indication of their pro-migratory propensity, and hence “cancerous” behaviour. Figure 1D shows that all PDAC cells have a cancer-type organisation of their cytoskeleton, with dendritic arrays of actin filaments.

When the cells were cultured as 3D, employing the U tube technique, PANC1 and MiaPaCa2 cells grew to form big spheroids, with a comparable growth rate over the 10 days’ culture period. On the other hand, BxPC3 showed very low or null growth rate (Fig. 2A). As better evident from Fig. 2B, the three different PDAC cells formed spheroids with varying degree of compactness: both PANC1 and MiaPaCa2 cells gave rise to big and loosely compact spheroids. PANC1 spheroids were also characterised by branches of cells coming out from the originary spheroid mass. Moreover, they showed a clear distinction between tightly formed round central dormant and loosely ruffled peripheral cells. On the contrary, BxPC3 formed very small and highly compact spheroids, with only few scattered cells outgrowing from the originary mass, with no distinction between central and peripheral region. Calcein/PI staining (Fig. 2C) showed that the majority of cells in the spheroid were viable after 72 h of incubation. Furthermore, the PDAC cells in the spheroids showed an actin cytoskeleton compactly arranged, with short actin filament distributed around the periphery of cells, especially where cell-cell interaction and aggregation took place (Fig. 2D).

**Figure 3:**
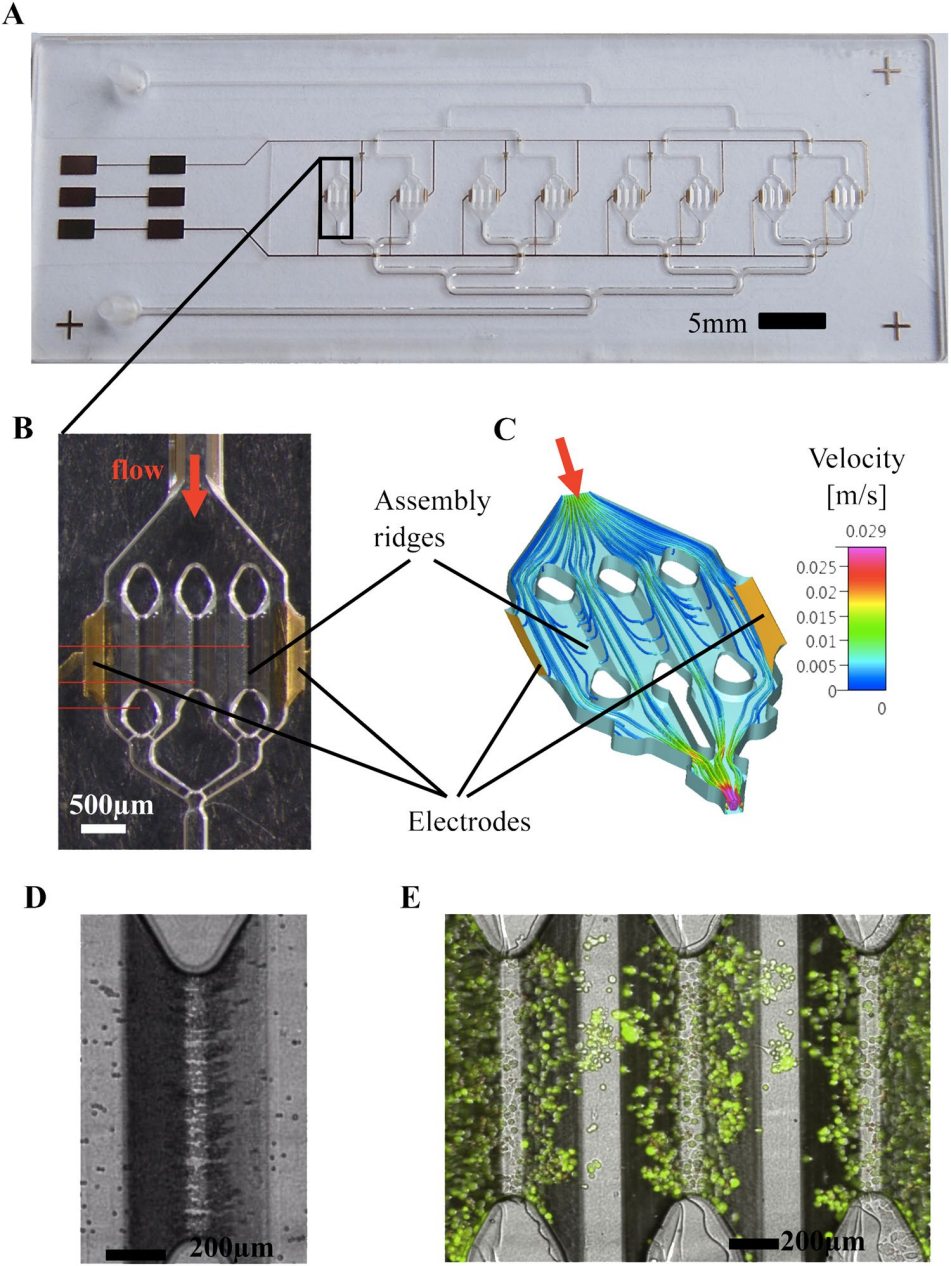
HepaChip: **(A)** Full view of the chip with 8 culture chambers, fluidic inlet and outlet and gold electrodes. **(B)** close up of one chamber containing 2 electrodes and 3 assembly ridges coated with collagen. **(C)** Simulation of flow velocity and trajectories of cells during DEP assembly inside a culture chamber. **(D)** PANC1 cells assembled on one assembly ridge right after assembly. **(E)** Live/Dead staining of PANC1 cells after 146 hours of perfused culture inside the HepaChip® chamber.

The same PDAC cell lines were then cultured in a novel type of microfluidic cell culture chamber, the HepaChip^®^. Figure 3A shows the HepaChip^®^, with fluid inlet and outlet, microchannels addressing eight cell chambers and electrodes contacting the lateral faces of each cell chamber (Fig. 3B). When energised with a high frequency (typically 100 kHz to 1 MHz) voltage, an inhomogeneous electric field is generated as local field strength is modulated by the assembly ridges positioned between opposite faces of the cell chambers. Under the media conditions chosen (low conductivity medium) cells are attracted from the cell suspension onto the assembly ridges. Figure 3C shows a multiphysics simulation of hydrodynamic and electrical forces acting on cells. Each line represents the calculated trajectory of a cell, part of which are ending on assembly ridges. Simulations as shown here enable assessment and optimisation of assembly efficiency for a given 3D microstructure of the chamber, voltage amplitude, and medium composition^14, 30^. A video of PANC1 cell assembly is provided as Supplementary Information (Supplementary Video S1). Figure 3D and E show typical examples of PANC1 cells on assembly ridges (immediately after assembly and after 146 h of culture under continuous perfusion, respectively).

**Figure 4:**
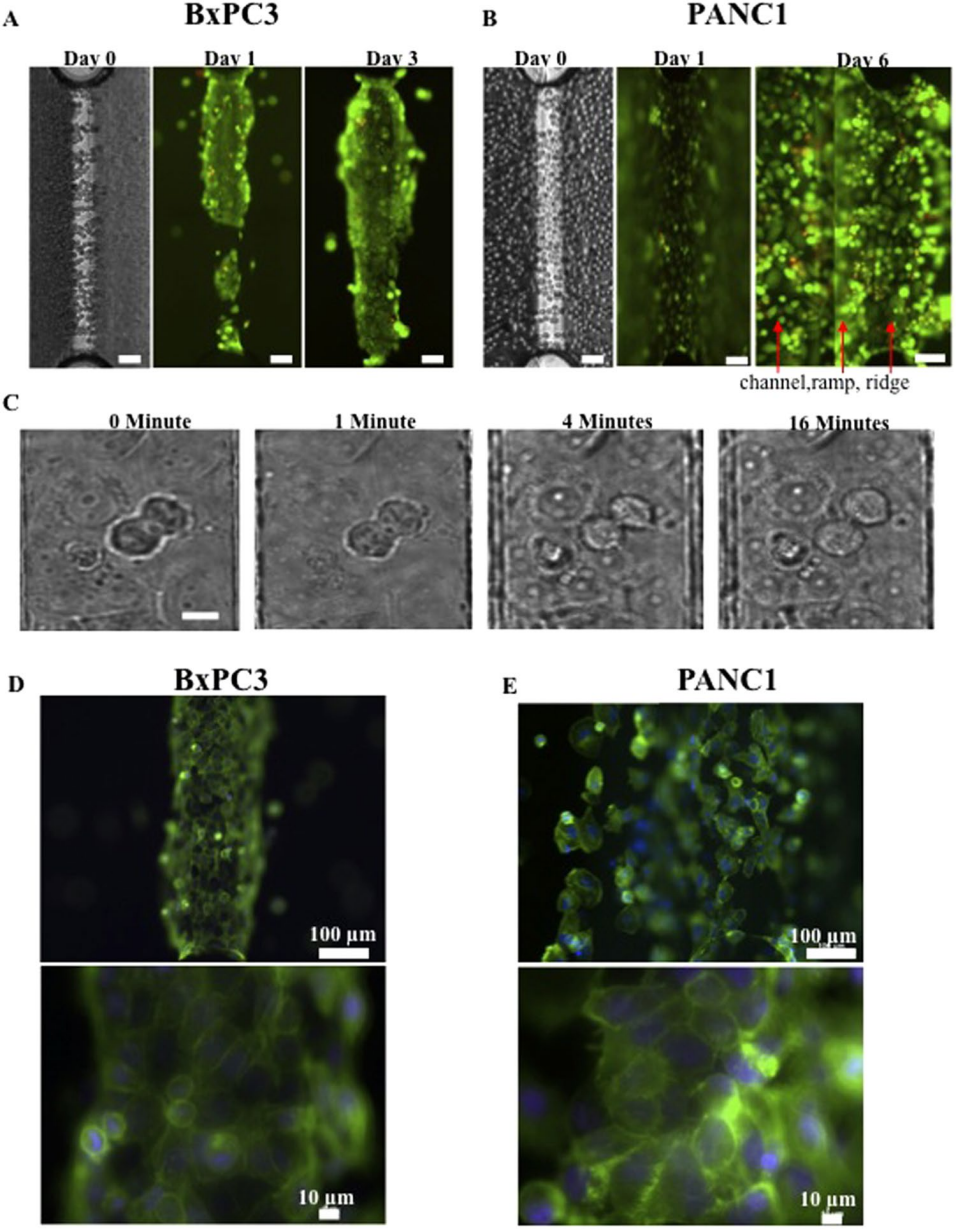
*In vitro* cell growth characteristics of PDAC cells in the HepaChip^®^. **(A)** Live/Dead cell staining of BxPC3: BxPC3 grow selectively on the ridge. **(B)** Live/Dead cell staining of PANC1: In contrast the PANC1 spread as well channel walls and bottom, so the picture on day 6 was taken in two focal planes: on the channel and on the ridge. **(C)** Mitosis of BxPC3: Mitosis was observed inside the chip on the collagen coated area after 16 h culture. From left to right pictures taken after 0 min – 1 min – 4 min – 16 min using an inverted microscope. **(D)** Cytoskeleton assembly of BxPC3 and **(E)** of PANC1: Fluorescence images after actin and nucleus staining with Phalloidin and DAPI. Scale bars in A and B represent 100 µm, in C represents 10 µm.

Viable BxPC3, MiaPaCa2 and PANC1 cells were assembled inside the HepaChip^®^ using DEP. When they were cultured inside the chip, all three cell types initially adhered to the chip surface. Both BxPC3 (Fig. 4A) and PANC1 (Fig. 4B) cells spread on the microstructure and remained viable for several days. Cells grew inside the HepaChip^®^ under perfusion with culture medium, as shown by the increase in cell number and density. We observed mitosis of both BxPC3 and PANC1 cells, as a clear index of their functionality. Figure 4C shows a representative example thereof obtained with BxPC3 cells. On the contrary, MiaPaCa2 cells showed very weak adhesion to the collagen-coated surfaces. This fact could be related to the expression, on the plasma membrane of these cells, of integrin adhesion receptors with lower affinity for collagen (Manoli S., personal communication). While some of them spread on the surface during the first 24 hours, others remained in a spherical shape and aggregated at columns and edges inside the chip. During the following three days of perfused culture the majority of MiaPaCa2 cells was transported out of the chip while vitality of the remaining cells decreased (see Supplementary Information Fig. S1).

Hence we focused our attention to BxPC3 and PANC1 cultures only (Fig. 4A and B). When comparing BxPC3 and PANC1 cultured within the microfluidic chip, we observed the following behaviours:

(1) **BxPC3**: during DEP BxPC3 cells adhered and spread on the collagen coated ridges until they formed a dense layer. During perfused culture of several days they slowly extended up the ramp and formed a thick rim. This rim grew thicker with culture time rather than further extending into the channel region (Fig. 4A). After 24 and 48 h, we observed mainly adherent, flattened cells, which continuously changed their shape. Furthermore, we observed small spheroids growing on top of the adherent cells.

(2) **PANC1**: during DEP PANC1 cells showed less adhesion to collagen coated areas but tended to slide over the surface before adhering. They were incubated for 10 to 15 min in DEP medium and left to sink onto the micro structured surface before the DEP medium was exchanged for culture medium. After that, PANC1 adhered everywhere in the chip, on collagen coating as well as on pluronic coated ramps of the ridges (Fig. 4B-0), on channel walls and on gold electrodes. They spread on any of these surfaces and grew one on top of others (Fig. 4 B-1 to B-6). In order to demonstrate the cell growth inside the 3D microstructure, Fig. 4B-6 was taken in two focal planes: that of the channel (left) as well as that of the collagen coated ridge (right). It shows that PANC1 also formed small aggregates on any of these surfaces after 6 days of cell culture. Overall, PANC1 cells behave inside the chip as a spheroid forming type. Actin stained with Phalloidin extended over the entire cytoplasm of both BxPC3 and PANC1 cells cultivated in the chip. As expected, it appeared denser along the cell membrane (Fig. 4D and E). PANC1 showed additional short actin filaments that extend from the membrane both into the cell and outside, a sign of the maintenance of their pro-migratory, cancerous features, even when cultured on the chip. The morphological and functional features of the three cell lines cultured as 2D, 3D and inside the chip are summarised in Table 1.

**Table 1:** Morphological and functional features of PDAC cells cultured in 2D, 3D and inside the microfluidics HepaChip device.

Culture type	Cell type	Adhesion	Spreading	Morphology	3D aggregate formation + aggregate size	Cytoskeleton
2D	BxPC3	Strong	Forms tight cell-cell clusters or islands of clusters with spread morphology	Epithelial	Forms large uniform clusters or islands of clusters.	Actin: Long and distributed across the cytoplasm
	PANC1	Moderate to strong	Spread morphology with few round shaped cells on top of spread cells	Epithelial	Forms moderate cell-cell aggregates	Actin: Long and distributed across the cytoplasm
	MiaPaCa2	Loose	Both round and spread shaped cells with few elongated feet like structures	Epithelial	Mostly separate cells with few cell-cell aggregates	Actin: Long and localised near the membrane level
3D	BxPC3	No adhesion to agarose		Smoothly tight small spheroid with no visible distinction between central and peripheral regions		Actin: Short and distributed around the periphery of the cell
	PANC1	No adhesion to agarose		Loosely assembled spheroid with clear distinction between tightly formed round central dormant and loosely ruffled (and proliferating) peripheral regions		Actin: Short and distributed around the periphery of the cell
	MiaPaCa2	No adhesion to agarose		Moderately tight spheroids with visible central dormant and peripheral proliferating regions		Actin: Short and distributed around the periphery of the cell
HepaChip	BxPC3	Selective on collagen	On collagen and cells	Mostly flat cells 20–30 µm diameter, few round 10 µm cells on top	At the edges of the coated microstructure w = 20–80 µm *	Actin: Long and distributed across the cytoplasm
	MiaPaca	Poor adhesion on collagen	Few cells spread on collagen, most stay spherical	Mostly spherical, 5–50 µm, after 24 h few flat cells with “feet”	Small aggregates at the edges of ridges and at pillars w = 80–90 µm*	
	PANC1	On microtopography independend of coating	On any surface and on top of other cells	Many flat cell with “feet”, various shapes, spherical ones on top, 10–50 µm	On the ramps w = 50–120 µm*	Actin: Long and distributed across the cytoplasm

^*^Maximum aggregate thickness in the HepaChip^®^ culture chamber was in the range of the measured width of the aggregates on the ramps. It is limited by the 3D geometry of the chamber between 40 µm on the assembly ridges and 190 µm inside the channels (see Fig. 3C) if assuming that at maximum aggregates can fill the height between the lid of the chamber and this ramp at the channel bottom.

**Figure 5:**
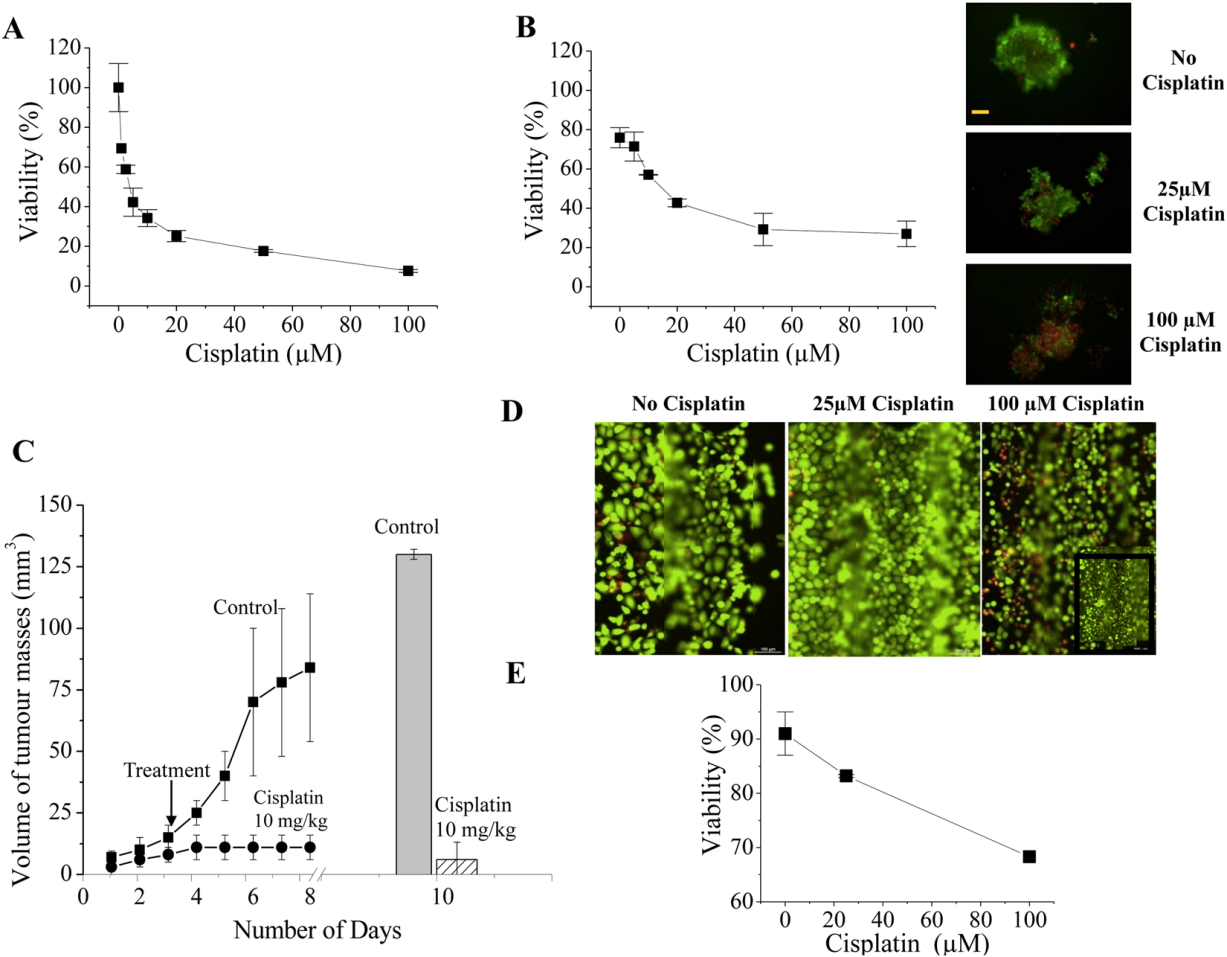
Effect of cisplatin on PANC1 cells. **(A)** Dose response curve of Cisplatin on PANC1 cells in 2D culture conditions. The calculated IC_50_ was 3.25 ± 0.2 μM. Data are means ± SD of two separate experiments, each carried out in triplicate. **(B)** Dose response curve of Cisplatin on PANC1 cells in 3D culture conditions. The calculated IC_50_ was 14.6 ± 1.6 μM. Data are means ± SD of two separate experiments, each carried out in triplicate. Panels on the right show the live/dead cell staining of the spheroids in control (panel on the top), 25 μM (panel in the middle), and 100 μM (panel at the bottom). **(C)** Effect of cisplatin on PANC1 cells subcutaneously injected into mice (*in vivo*). The trend line shows the volume of the masses during the duration of experiment, histogram on the right shows the tumour masses of explant from the animals. **(D)** Effect of cisplatin on PANC1 cells inside the HepaChip^®^ under continuous perfusion of 3 µL/min. Live/Dead cell staining after 72 h incubation with 0 µM, 25 µM and 100 µM cisplatin; inset: black framed area of the chip cell culture before incubation with 100 µM Cisplatin. Scale bars 100 µm. **(E)** Effect of different cisplatin concentrations on PANC1 cells cultured inside the HepaChip^®^.

Finally, we tested whether the microfluidic chip could be a useful device for testing drugs in PDAC cells. In particular, we tested the effects of Cisplatin, a widely used drug for many cancer types including PDAC, on PANC1 cells. Cisplatin exerted cytotoxic activity on PANC1, with IC_50_ values of 3.25 ± 0.2 μM (n = 2) and 14.6 ± 1.6 μM (n = 2) in 2D and 3D cultures, respectively. In 3D cultures 100 μM Cisplatin caused a decrease in cell viability (roughly 80%) after 72 h of incubation, and caused the spheroids to disaggregate after 72 h of treatment (Fig. 5B). We also tested the effects of Cisplatin *in vivo*, in immunocompromised mice subcutaneously injected with PANC1 cells, at 10 mg/kg^31^. As expected, this Cisplatin dose fully blocked tumour growth after 10 days (Fig. 5C). The effects of Cisplatin on PANC1 cells cultured inside the microfluidic chamber were also analysed (Fig. 5D). After 72 h of perfusion with 25 µM Cisplatin in culture medium, a certain reduction of viability from 95 to 83% in both chips and a slight decrease of cell density were observed. The highest concentration of 100 µM Cisplatin perfused through the cell culture chip started to reduce viability from 92 to 85% after 24 h incubation, reaching 68% after 3 days. At this time an overall reduction of the cell density in the chip was observed as shown in the yellow framed area of Fig. 5D on the right compared to the inset below that shows the same area of this chip before incubation with Cisplatin. Viabilities in the control chips without Cisplatin remained between 90 and 78% over a culture time of 5 days in total. Overall a trend in concentration dependence of Cisplatin effects is evident in the graph in Fig. 5E.

The higher Cisplatin concentration needed to obtain reduction in cell vitality in the chip compared to conventional 2D *in vitro* cultures could be due either to differences in cellular functions (e.g. presence of a structured tridimensional ECM in the chip) or to an unfavourable adsorption of the drug onto the microfluidic device which might then result in a lower effective concentration acting on the cells. To test this latter hypothesis, and taking into account the fact that Cisplatin is relatively hydrophobic, we tested the recovery of a number of compounds covering a wide range with respect to hydrophobicity. Uncoated chips showed a considerable degree of adsorption, so that that the most hydrophobic compounds tested (Bupropion, Amodiaquine) were recovered at below 50% even after 3 hours of perfusion. On the contrary, coated chips identical to those used in this study, exhibited almost complete recovery after only 1 hour of perfusion (Supplementary Information Fig. S2). Therefore, considering that Cisplatin was applied over a period of 72 h, adsorption to polymer surfaces can be ruled out as the cause for the high doses of the drug needed to affect viability of PANC1 cells.

## Discussion

In the present paper we examined the possibility of using a microfluidic platform suitable for studying biomolecular characteristics of PDAC cells. Such platform could be further employed to improve diagnosis and prognosis of PDAC as well as for personalised pharmacological testing, in the future. We chose to study PDAC cells since PDAC is one of the human cancers with worst prognosis, with a 5-year survival rate less than 6%. PDAC malignancy can be traced back to the limited capabilities to diagnose (and treat) the disease in its earliest stages, its propensity to migrate and invade surrounding tissues and hence give rise to local and distant metastases, as well as to its substantial chemo- and radio-resistance. Overall new strategies are urgently needed to impact PDAC treatment and to extend and save PDAC patients’ lives. For these purposes, various cancer model systems^26^ have been developed, from *in vitro* 2D and 3D cell cultures to whole animal models.

We here provide evidence that PDAC cells can be cultured onto a novel microfluidic chamber, the HepaChip^®^, maintaining cell vitality, morphological appearance and growth characteristics that more closely resemble 3D cultures. Furthermore the actin organisation, taken as a functional marker of a proper pro-migratory, neoplastic phenotype, showed a typical cancer-type assembly. Several cultures of PDAC cells in microfluidic channels have been published that culture one or more cell types entrapped in a hydrogel scaffold containing ECM proteins^26, 32^. Although the microfluidic structures used so far, allow microscopic image acquisition similar to 2D *in vitro* assays^32, 33^, they do not mimic the flow of nutrients as occurs inside blood vessels and around them. Such a flow produces shear forces and pressure, which closely resemble *in vivo* tissues^34, 35^. The microfluidic system (HepaChip^®^) we have developed allows for continuous perfusion of the cell culture. Furthermore, viable cells are actively assembled in a dense layer inside the HepaChip^®^ culture chamber without entrapping them inside a hydrogel that might slow down the transport of nutrients to and metabolites from the cells. The 3D structure of the HepaChip^®^ enables the formation of cell aggregates especially in areas of reduced flow between the pillars. These aggregates are still in direct contact to the medium flow and hence delivered a continuously high concentration of nutrients. Media perfused through the chip was assumed to be saturated by oxygen upon entering the chip as the tubing employed was permeable to oxygen. At a perfusion rate of 3.125 µl/min oxygen was supplied at a rate of 1.2 nmol/min to the chip, by far in line with common oxygen perfusion and pressure inside pancreatic cancers *in vivo* ^36^. This means they can also be exposed to a continuously high dose of drug during toxicity tests.

Indeed, we also tested the suitability of the platform for testing chemotherapeutic drugs. We chose to test Cisplatin as a model drug, since Platinum-based drugs are used in both the neo-adjuvant and the adjuvant settings for PDAC treatment. One major hindrance when testing chemotherapeutic drugs *in vivo* (both in preclinical models and in patients) is the frequent lack of response applying drug concentrations derived from *in vitro* data, obtained in standard 2D cultures^37–39^. We confirmed these findings showing that the IC_50_s of Cisplatin in PANC1 cells cultured *in vitro* are around 3 μM in 2D cultures, and only slightly higher, 15 μM, in spheroid 3D cultures. On the contrary, the dose used to obtain complete growth inhibition (10 mg/kg) *in vivo* is much higher, corresponding to an estimated plasma concentration of 240 μM^40^. When tested on cells cultured onto the chip, Cisplatin decreased PANC1 cell vitality (about 30%) after 72 hours of incubation with high doses (100 μM). This dose is much higher than the IC_50_s determined in *in vitro* 2D and 3D cultures. Although these are still singular experiments and a dose-response assay was not performed, the necessity of higher doses of a chemotherapeutic drug to decrease cell viability when applied to a chip are consistent with those previously reported^32, 33^. Moreover, although we cannot compare the results obtained in the chip with those found in mice, our data suggest that the effects of Cisplatin perfused in the chip are more similar to those exerted *in vivo*, e.g. in mice (Fig. 5C). This is consistent with data reported in^41^ showing that the IC_50_ values of five model drugs, tested in a microfluidic chip for *in vitro* hepatocyte drug toxicity tests, correlate with the IC_50_s determined *in vivo*. Overall our data suggest that PDAC cells, when cultured in the microfluidic chip under continuous perfusion, withstand incubation with Cisplatin at higher doses compared to classical *in vitro* cultures. Our data showing no adsorption of the drug to polymer surfaces, lead to exclude drug adsorption as the cause for this chemo- resistance. The proper culture in the chip needs cell adhesion to collagen, that can re-establish the cell-cell and cell-ECM interactions that mimic the real tissues^42^. Hence we hypothesise that when PDAC cells are cultured in the chip behave differently compared to 2D. For example they could form a thin layer of ECM around them under flow conditions which could shield from perfused compounds. Consistently, the ECM is known to sustain tumour chemoresistance^43^, through the triggering of different molecular mechanism, including an up regulation of the Multi Drug Resistance p-glycoprotein^44^. Although we will deepen these points in the future, from the present paper it appears that tumour cell cultures onto the HepaChip^®^ provide a more realistic and predictive response towards chemotherapeutic agents when compared to 2D cultures of the same cell type.

Overall, we provide a novel microfluidic platform that could be fruitfully transferred to both clinical and pharmaceutical testing, in order to provide biological characteristics as well as responses to drug treatments, to improve a personalised approach to PDAC.

## Electronic supplementary material

Supplementary Informations

Supplementary Video S1

## References

[1] Herreros-Villanueva M, Hijona E, Cosme A, Bujanda L (2012). Mouse models of pancreatic cancer. World J. Gastroenterol..

[2] Feldmann G, Rauenzahn S, Maitra A (2009). *In vitro* models of pancreatic cancer for translational oncology research. Expert Opin Drug Discov.

[3] Coleman SJ (2014). Pancreatic cancer organotypics: High throughput, preclinical models for pharmacological agent evaluation. World J. Gastroenterol..

[4] Edmondson R, Broglie JJ, Adcock AF, Yang L (2014). Three-dimensional cell culture systems and their applications in drug discovery and cell-based biosensors. Assay Drug Dev Technol.

[5] Breslin S, O’Driscoll L (2013). Three-dimensional cell culture: the missing link in drug discovery. Drug Discov. Today.

[6] Trédan O, Galmarini CM, Patel K, Tannock IF (2007). Drug resistance and the solid tumor microenvironment. J. Natl. Cancer Inst..

[7] Hirschhaeuser F (2010). Multicellular tumor spheroids: an underestimated tool is catching up again. J. Biotechnol..

[8] Santini MT, Rainaldi G, Indovina PL (2000). Apoptosis, cell adhesion and the extracellular matrix in the three-dimensional growth of multicellular tumor spheroids. Crit. Rev. Oncol. Hematol.

[9] Elliott NT, Yuan F (2011). A review of three-dimensional *in vitro* tissue models for drug discovery and transport studies. J Pharm Sci.

[10] Astashkina A, Grainger DW (2014). Critical analysis of 3-D organoid *in vitro* cell culture models for high-throughput drug candidate toxicity assessments. Adv. Drug Deliv. Rev..

[11] Huh D, Hamilton GA, Ingber DE (2011). From 3D cell culture to organs-on-chips. Trends Cell Biol.

[12] Verhulsel M (2014). A review of microfabrication and hydrogel engineering for micro-organs on chips. Biomaterials.

[13] Esch EW, Bahinski A, Huh D (2015). Organs-on-chips at the frontiers of drug discovery. Nat Rev Drug Discov.

[14] Huh D, Torisawa YS, Hamilton GA, Kim HJ, Ingber DE (2012). Microengineered physiological biomimicry: organs-on-chips. Lab Chip.

[15] Boussommier-Calleja A, Li R, Chen MB, Wong SC, Kamm RD (2016). Microfluidics: A new tool for modeling cancer-immune interactions. Trends Cancer.

[16] Dickinson LE, Lütgebaucks C, Lewis DM, Gerecht S (2012). Patterning microscale extracellular matrices to study endothelial and cancer cell interactions *in vitro*. Lab Chip.

[17] Seo BR, Delnero P, Fischbach C (2014). *In vitro* models of tumor vessels and matrix: engineering approaches to investigate transport limitations and drug delivery in cancer. Adv. Drug Deliv. Rev..

[18] Vidi P-AA (2014). Disease-on-a-chip: mimicry of tumor growth in mammary ducts. Lab Chip.

[19] Yates C (2007). Novel three-dimensional organotypic liver bioreactor to directly visualize early events in metastatic progression. Adv. Cancer Res.

[20] Zhang Y (2015). [comparisons of pharmacokinetic profile of eleven bioactive components in Haizao Yuhu decoction modified with Haizao and Gancao anti-drug pair in normal rats]. Zhongguo Zhong Yao Za Zhi.

[21] Schütte J (2011). ‘Artificial micro organs’–a microfluidic device for dielectrophoretic assembly of liver sinusoids. Biomed Microdevices.

[22] Ho C-TT, Lin R-ZZ, Chang W-YY, Chang H-YY, Liu C-HH (2006). Rapid heterogeneous liver-cell on-chip patterning via the enhanced field-induced dielectrophoresis trap. Lab Chip.

[23] Hagmeyer B, Zechnall F, Stelzle M (2014). Towards plug and play filling of microfluidic devices by utilizing networks of capillary stop valves. Biomicrofluidics.

[24] Holzner F (2011). Numerical modelling and measurement of cell trajectories in 3-D under the influence of dielectrophoretic and hydrodynamic forces. Electrophoresis.

[25] Schütte J (2010). A method for patterned in situ biofunctionalization in injection-molded microfluidic devices. Lab Chip.

[26] Hwang C-II, Boj SF, Clevers H, Tuveson DA (2016). Preclinical models of pancreatic ductal adenocarcinoma. J. Pathol..

[27] Deer EL (2010). Phenotype and genotype of pancreatic cancer cell lines. Pancreas.

[28] Chen W (2014). High-throughput image analysis of tumor spheroids: a user-friendly software application to measure the size of spheroids automatically and accurately. J Vis Exp.

[29] Yamaguchi H, Condeelis J (2007). Regulation of the actin cytoskeleton in cancer cell migration and invasion. Biochim. Biophys. Acta.

[30] Chiu DT (2000). Patterned deposition of cells and proteins onto surfaces by using three-dimensional microfluidic systems. Proc. Natl. Acad. Sci. USA.

[31] Fujiwara M (2008). Modulating effect of the PI3-kinase inhibitor LY294002 on cisplatin in human pancreatic cancer cells. J. Exp. Clin. Cancer Res..

[32] Lovitt CJ, Shelper TB, Avery VM (2014). Advanced cell culture techniques for cancer drug discovery. Biology (Basel).

[33] Drifka CR, Eliceiri KW, Weber SM, Kao WJ (2013). A bioengineered heterotypic stroma-cancer microenvironment model to study pancreatic ductal adenocarcinoma. Lab Chip.

[34] Herrmann D (2014). Three-dimensional cancer models mimic cell-matrix interactions in the tumour microenvironment. Carcinogenesis.

[35] Seo BR, Delnero P, Fischbach C (2014). *In vitro* models of tumor vessels and matrix: engineering approaches to investigate transport limitations and drug delivery in cancer. Adv. Drug Deliv. Rev..

[36] Koong AC, Mehta VK, Le QT, Fisher GA, Terris DJ, Brown JM, Bastidas AJ, Vierra M (2000). Pancreatic tumors show high levels of hypoxia. Int. J. Radiation Oncology Biol. Phys..

[37] Denayer T, Stöhr T, Roy M (2014). Animal models in translational medicine: Validation and prediction. New Horizons Transl Medicine.

[38] Heinemann V (2006). Randomized phase III trial of gemcitabine plus cisplatin compared with gemcitabine alone in advanced pancreatic cancer. J. Clin. Oncol..

[39] Hwang IG (2012). A phase II trial of Erlotinib in combination with gemcitabine and cisplatin in advanced pancreatic cancer. Invest New Drugs.

[40] Hennik MBvan (1987). Comparative pharmacokinetics of cisplatin and three analogues in mice and humans. Cancer Res..

[41] Toh Y-CC (2009). A microfluidic 3D hepatocyte chip for drug toxicity testing. Lab Chip.

[42] Pampaloni F, Reynaud EG, Stelzer EH (2007). The third dimension bridges the gap between cell culture and live tissue. Nat. Rev. Mol. Cell Biol..

[43] Pillozzi S (2011). Chemotherapy resistance in acute lymphoblastic leukemia requires hERG1 channels and is overcome by hERG1 blockers. Blood.

[44] Xu H (2016). Development of three-dimensional breast cancer cell culture drug resistance model. Sheng Li Xue Bao.

