# Supplementary material for: A novel microfluidic 3D platform for culturing pancreatic ductal adenocarcinoma cells: comparison with in vitro cultures and in vivo xenografts
Source: Sci Rep. 2017 Apr 25;7:1325. doi: 10.1038/s41598-017-01256-8 (PMC5430997; doi:10.1038/s41598-017-01256-8)

Supplementary Informations

A novel microfluidic 3D platform for culturing pancreatic ductal adenocarcinoma cells: comparison with *in vitro* cultures and *in vivo* xenografts.

Meike Beer[1°], Nirmala Kuppalu[2°], Matteo Stefanini[3], Holger Becker[4], Ingo Schulz[4], Sagar Manoli[2], Julia Schuette[1], Christian Schmees[1], Armando Casazza[3], Martin Stelzle[1°] and Annarosa Arcangeli[2°*]

Supplementary Video S1.

Microscopic video of PANC1 cells assembled in the HepaChip using dielectrophoresis at 350 kHz and 145 V peak to peak at a flow rate of 100 µL/min. The cells assemble in pearl chains on the assembly ridges inside the culture chamber due to the dielectrophoretic force and adhere on the collagen coating in these areas.

Supplementary Figure S1: MiaPaCa2 in the HepaChip directly after assembly using DEP after 5,21,69 and 91 h of cell culture under perfusion; scale bars 100µm.

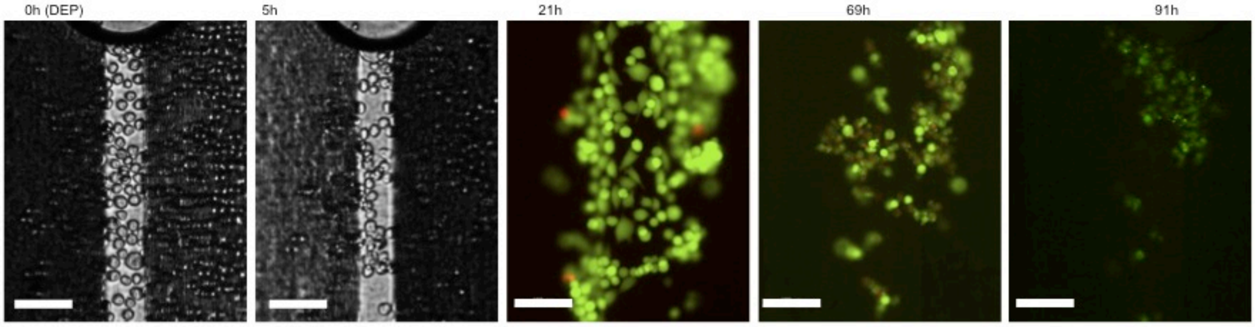

Supplementary Figure S2: Recovery of different compounds from uncoated and coated polymer

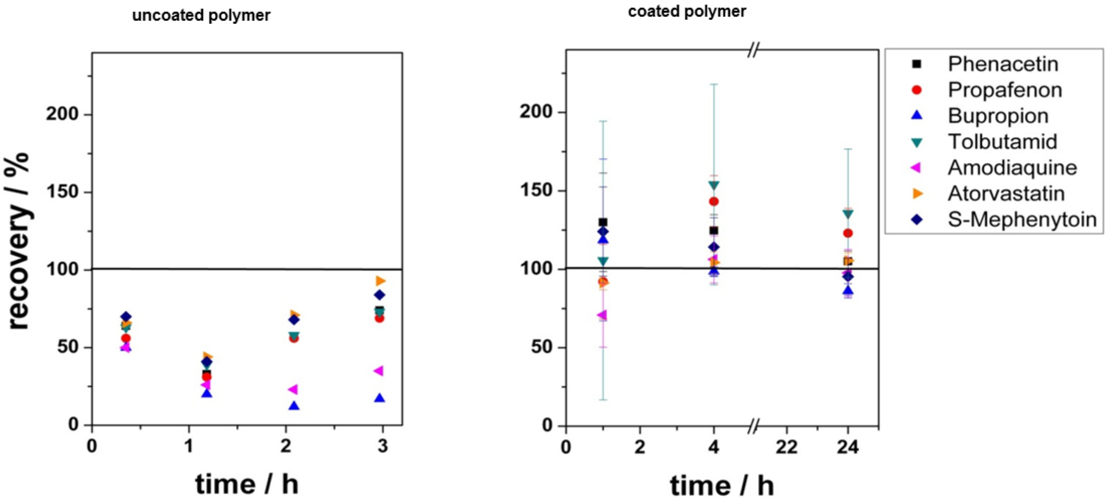

Supplement: Supplementary file 1 — Supplementary Informations [file 41598_2017_1256_MOESM1_ESM.pdf]
